# In Vitro and In Vivo Effect of 5-FC Combined Gene Therapy with TNF-α and CD Suicide Gene on Human Laryngeal Carcinoma Cell Line Hep-2

**DOI:** 10.1371/journal.pone.0061136

**Published:** 2013-04-11

**Authors:** Li-Ping Chai, Zhang-Feng Wang, Wei-Ying Liang, Lei Chen, Dan Chen, An-Xun Wang, Zhao-Qiang Zhang

**Affiliations:** 1 Department of Otolaryngology, First Affiliated Hospital and Otolaryngology Institute, Sun Yat-Sen University, Guangzhou, Guangdong, P. R. China; 2 Department of Otolaryngology, The University of Hong Kong-Shenzhen Hospital, Shenzhen, Guangdong Province, P. R. China; 3 Department of Burns Surgery, First Affiliated Hospital of Sun Yat-sen University, Guangzhou, Guangdong, P. R. China; 4 Department of Oral and Mallofacial Surgery, First Affiliated Hospital of Sun Yat-Sen University, Guangzhou, Guangdong, P. R. China; 5 Department of Oral and Maxillofacial Surgery, Hospital of Stomatology, Guangzhou Medical University, Guangzhou, Guangdong, P. R. China; The University of Hong Kong, China

## Abstract

This study was aimed to investigate the effect of combined cancer gene therapy with exogenous tumor necrosis factor-alpha (TNF-α) and cytosine deaminase (CD) suicide gene on laryngeal carcinoma cell line Hep-2 *in vitro* and *in vivo*. Transfection of the recombinant eukaryotic vectors of pcDNA3.1 (+) containing TNF-α and/or CD into Hep-2 cells resulted in expression of TNF-α and/or CD gene *in vitro*. The significant increase in apoptotic Hep-2 cells and decrease of Hep-2 cell proliferation were observed using 5-FC treatment combined with TNF-a expression by CD/5-FC suicide system. Moreover, bystander effect was also observed in the TNF-α and CD gene co-expression group. Laryngeal squamous cell carcinoma (LSCC) mice model was established by using BALB/c mice which different transfected Hep-2 cells with pcDNA3.1 (+) containing TNF-α and/or CD were applied subcutaneously. So these mice are divided into four groups, namely, 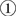
Hep-2/TIC group; 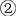
Hep-2/CD group; 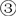
Hep-2/TNF-α group; 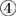
Hep-2/0 group. At day 29 after cell inoculation, volume of grafted tumor had significant difference between each two of them (*P*<0.05). These results showed that the products of combined CD and TNF-α genes inhibited the growth of transplanted LSCC in mice model. So by our observed parameters and many others results, we hypothesized that 5-FC combined gene therapy with TNF-αand CD suicide gene should be an effective treatment on Laryngeal carcinoma.

## Introduction

Laryngeal carcinoma (LC) is one of the most prevalent malignant tumors in the head and neck area. Approximately a global population of 190, 000 people are diagnosed this disease per year [1], more importantly, the number is increasing year by year [2]–[3]. Therefore, LC is still the major cause of cancer-related death which owns significant threat on human's health on a worldwide scale. Since 1873, after Billroth performed the very first total laryngectomy, surgical treatment of LC has been developing for more than 100 years. However, until now, advanced LC treatment is still a huge challenge. With surgery and post-surgical adjuvant radiotherapy or chemotherapy, only less than 60% of the patients achieved 5-year survival [4]–[6]. In addition, surgery might lead to complete or partial loss of swallowing and vocal function, so many patients have to maintain a tracheal cannula on a long-term basis due to laryngeal stenosis after surgery; such problems have impaired their quality of life remarkably [7] Thus we are seeking for a promising treatment strategy for the treatment of middle and advanced stage of LC. In addition, a promising treatment strategy should ensure treatment efficacy, reduce treatment-related toxicity reaction and improve quality of life. More importantly, these aspects have been climbed into the top priority consideration. A large variety of therapeutic genes such as tumor suppressor, antiangiogenesis, suicide and micro-RNA gene will be under our investigation. Tumor Necrosis Factor-alpha (TNF-α) can inhibit tumor progress through different signal pathways, such as cells apoptosis, killing cells by direct cytotoxicity effect or damaging the microvasculature of tumor tissue. In 1990, Rosenberg used TNF-α gene transfected (as mediated by retrovirus) tumor infiltrating lymphocyte (TIL) to treat patients with advanced malignant melanoma and revealed remission to varied extents in these patients [8]. The enzyme/prodrug system of cytosine deaminase/5-fluorocytosine (CD/5-FC) enzyme/prodrug combination, found in the year of 1992 [9], is an gene therapy approach received considerable attention followed with another enzyme/prodrug system, herpes simplex virus thymidine kinase/ganciclovir(HSV-tk/GCV) combination. Mechanisms of CD/5-FC system is that CD specifically converts the antifungal agent 5-FC to the highly toxic 5-FU, which exerts its toxic effect by imparing DNA biosynthesis, resulting in the death of aimed cells. TNF-α is known for as the strongest antitumor cytokine in the current time, plays a key role in killing tumor cells and in hemorrhagic necrosis as well as in immunity. However, the effectiveness of TNF-α is limited by the systemic toxicity associated with clinical treatment. In addition, currently, there is no research which has been reported using CD gene combined with TNF-α gene in treating cancer. Based on these above background, we hypothesis that, by transfectting eukaryotic vector pcDNA3.1 (+) carrying suicide CD and TNF-α gene into laryngeal cancer cell-Hep-2, this method can combine the complementary advantage of each gene, thereby improve the efficacy of gene therapy, increase anti-tumor immune responses, reduce their toxicity, exert the synergic cytocidal effect of suicidal gene as well as cytokine gene on tumor cells.

### Ethics statement

This study was approved by the Ethics Committee of First Affiliated Hospital of Sun Yat-Sen University. All efforts were made to minimize mice suffering. All mice were killed by cervical dislocation and decapitations and all surgeries were performed under anesthesia with urethane (2 mg/kg).

## Materials and Methods

### Plasmids, strains and cell line

Escherichia coli JM109, transformed recipient bacteria *E*coliDH-5α, plasmid pcDNA3.1 (+) and plasmid pIRES were preserved in the Biochemical Laboratory of Sun Yat-Sen University. Human laryngeal carcinoma cell line Hep-2 was purchased from Shanghai Cell Bank, Chinese Academy of Science. The human laryngeal carcinoma cell Hep-2 was incubated in DMEM medium containing 10% fetal bovine serum at 5% CO_2_ and 37°C.

### Animals

Weighing 18–22 g, 56 SPF BALB/c nu/nu nude mice (28 male and 28 female) aged 4–6 weeks were purchased from Animal Center (certificate number: SCK (Yue) 2003-0001 Yue regulatory certificate number 2007A015), Guangzhou University of Chinese Medicine. They were then kept and used for the experiments in the Laboratory Animal Center of Sun Yat-Sen University in Guangzhou (certificate number: Yue SYXK 2007-0081, serial number 0025415, 0027935 and 0028045).

### Cloning of target genes and construction of vector

According to the nucleotide sequence of TNF-α gene (access number: U77396) and CD gene (access number: AY331712) provided in GenBank, EcoR Ι and Not Ι restriction enzyme sites were inserted into up-stream and down-stream of TNF-α gene; Xho Ι and Apa Ι restriction enzyme sites were inserted into the 5′ terminal of the up-stream and down-stream primers of CD gene. Initiation codon and termination codon that agreed with the reading frame were also introduced. PCR primers were designed in accordance to the designing principles and were shown in **table 1**. Primers were constructed by Shanghai Sangon Biological Engineering Technology & Service Co., Ltd. Genomic DNA of Escherichia coli JM 109 strain was used as template for the PCR amplification. Genomic DNA of the bacteria was extracted by the standard method described in *Molecular Cloning* [10] Total RNA was extracted from human peripheral white blood cells and underwent RT-PCR for the amplification of TNF-α gene. Expression vectors pcDNA 3.1(+)-CD and pcDNA 3.1(+)-TNF-α were constructed by forwardly inserting target genes TNF-α gene or CD gene into the cloning site of Xho Ι/Apa Ι or EcoR Ι/Not Ι in the plasmid pcDNA 3.1(+), respectively. PIRES plasmid was cleavaged by double restriction enzyme of Not Ι and Xho Ι, and thereby IRES segment was extracted by gel extraction. Plasmid pcDNA 3.1(+)-CD was cleavaged by double restriction enzymes of EcoR Ι and Not Ι, then TNF-α segment was inserted to construct the plasmid pcDNA 3.1(+)-TNF-α-CD. Such plasmid was again cleavaged by double enzymes of Not Ι and Xho Ι and inserted with IRES segment to construct the expression vector pcDNA 3.1(+)-TNF-α-IRES-CD.

**Table 1:** Sequence of the primers and size of amplified segments.

Gene name	Sequence of primer and site of segment	Size
CD	Forward: 5′-gg ctcgagt atg tcg aat aac gct tta ca-3′	1284 bp
	Reverse: 5′-cc gggccc tca acg ttt gta atc gat gg-3′	
TNF	Forward: 5′-gg gaattc atg gcg gtt cca gga cct tac-3′	687 bp
	Reverse: 5′-gc gcggccgc cta tgc acg act cca agc ag-3′	

### Gene transfection

Liposome-mediated gene transfection was performed as the protocol of Lipofectamine 2000. Hep-2 cells were transfected by the plasmids pcDNA 3.1(+)-TNF-α-CD, pcDNA3.1(+)-CD, pcDNA 3.1(+)-TNF-α and pcDNA3.1(+) (blank control), respectively. At 2 days after the transfection, cells were incubated with 600μg/ml G418 for additional two weeks. Positive clones were obtained, amplified and named as Hep-2/TIC, Hep-2/CD, Hep-2/TNF-α and Hep-2/0, respectively.

### Gene expression verification by RT-PCR

Total RNA was extracted from Hep-2/TIC, Hep-2/CD, Hep-2/TNF-α and Hep-2/0 cells by guanidinum-phenol-chloroform method. Primers were designed according to the gene sequences published in GenBank (**Table 2**) and were constructed by Shanghai Sangon Biological Technology & Service Co., Ltd. After reverse transcription, PCR reactions were catalyzed. PRC products then underwent gel electrophoresis.

**Table 2:** Sequence of primers and size of amplified segments.

Gene name	Sequence of primer and site of segment	Site
CD	Forward: 5'-ata tcc agc aca gtg gcg gc-3'	340 bp
	Reverse: 5'-gcg act tcc tgc ttc act tc-3'	
TNF	Forward: 5'-ctg ctg ctt cat ccc ctt ct-3'	273 bp
	Reverse: 5'-ttc tgg ggt ttg gag att tg-3'	

### In vitro experiments on cytocidal effect

A number of 5×10^3^ Hep-2/TIC, Hep-2/CD, Hep-2/TNF-α, Hep-2/0 and Hep-2 parent cells were inoculated into 96-well plate, with 18 wells for each type of cells. The plate was then incubated at 37°C and 5%CO_2_ for 24 hours. 5-FC was then added at the concentration level of 0, 0.001, 0.01, 0.1, 1 and 10 mmol/L, with 3 wells for each concentration level. The cells were then incubated for another 96 hours and were added with MTT, in which the cells were cultivated for another 4 hours. Supernatant was then removed and Dimethyl Sulfoxide (DMSO) was added. OD value at 570 nm was detected by ELISA reader. Cell survival rate was calculated by: survival rate(%) = A/B×100% (A: OD value of Hep-2/TIC, Hep-2/CD, Hep-2/TNF-α or Hep-2/0cells; B: OD value of Hep-2 parent cells).

A total of three repeated experiments were performed. Survival curve was generated with mean survival rates as ordinate axis and concentration levels of 5-FC as abscissa axis.

### Bystander effect

The bystander effect refers to the induction of biological effects in cells that are not directly traversed by a charged particle [11]. By-stander effect was observed in Hep-2/TIC, Hep-2/CD, Hep-2/TNF-α, Hep-2/0 and un-transfected Hep-2 parent cells. A total of 5×10^3^ mixed cells with varied percentages of parent cells and each type of transfected cells (each transfected cells could account for 0%, 10%, 20%, 50% and 100% of total cells) were inoculated onto 96-well plate, with 3 wells for each cell mixture. At 24 hours later, 1 mmol/L 5-FC was added. After being incubated for 96 hours, the cells were added with MTT. Another 4 hours later, supernatant was removed and DMSO was added. OD value at 570 nm was detected by ELISA reader. Cell survival rate was detected by 3 repeated measurements.

### Analysis on apoptosis

Apoptotic rates in Hep-2/TIC, Hep-2/CD, Hep-2/TNF-α and Hep-2/0 cells when treated by 1 mmol/L 5-FC were detected by flow cytometer (Prodidium Iodide Staining). A total of 5×10^3^ cells of each type were inoculated into 96-well plate and were incubated at 37°C and 5%CO_2_ for 24 hours. Then 1 mmol/L 5-FC was added and the cells were incubated for the next 96 hours. Then cells were prepared into single cell suspension. The cell concentration was maintained at 5×10^5^–1×10^6^ cells/ml, with 3 tubes for each type of cells. The cell suspension was then sent to the First Affiliated Hospital, Sun Yat-Sen University for flow cytometry.

### Detection of TNF-α in the supernatant of in vitro cell culture

A total of 5×10^3^ Hep-2/TIC, Hep-2/CD, Hep-2/TNF-α and Hep-2/0 cells were inoculated into 96-well plate, which was then incubated at 37°C and 5%CO_2_ for 50 hours. Supernatant was obtained from each well and was divided, with 100 <mu>l in each portion. According to the protocol of TNF-α Elisa detection reagent kit (Jingmei Biotech Company of Beijing, China), standard wells of 125 pg/ml, 62.50 pg/ml, 31.25 pg/ml, 15.65 pg/ml, 7.8 pg/ml and 0 pg/ml and blank well were prepared; experimental wells were also prepared using the supernatant of each cell culture, with 3 wells for each type of cells. OD value at 570 nm was detected by ELISA reader. Standard curve was generated and OD values of experiment samples were obtained using the standard curve.

### Establishment of subcutaneous grafted tumor model of human laryngeal carcinoma in nude mice

Tumor cell suspension was inoculated into 53 BALB/c nu/nu nude mice. The nude mice were randomly assigned into 4 groups. Experiment groups: 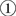
Hep-2/TIC group: 13 nude mice; 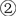
Hep-2/CD group: 13 nude mice; 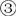
Hep-2/TNF-α group: 13 nude mice; control group: 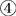
Hep-2/0 group,14 nude mice. Hep-2/TIC, Hep-2/CD, Hep-2/TNF-α and Hep-2/0 cells at logarithmic growth phase were subcutaneously inoculated into the lateral skin of armpit of the right forearm, so as to establish subcutaneous grafted tumor models in nude mice. The cell suspension was prepared by suspending 5×10^6^ cells of each type into 0.2 mL PBS.

### Drug administration in nude mice and observed parameters

Treatment started at 8 days after cell inoculation. For Hep-2/TIC, Hep-2/CD and Hep-2/TNF-α group, 5-FC at the dosage of 500 mg· kg^−1^·d^−1^ was given by intraperitoneal injection everyday (0.25 ml/time, twice a day); for Hep-2/0 group: the same amount of NS was injected. The treatment lasted for 12 lasted days. Every 3 days, vernier caliper was used to measure the size of the tumor (maximum major diameter and transverse diameter) and tumor volume was calculated by the formula: V = 1/2ab^2^. Tumor growth curve was plotted. Curve fitting equation was V = A*e* ^k t^. Tumor doubling time was calculated by *T* = ln2/*K* (k: growth rate). Based on the mean tumor volume of each group, tumor growth curve was generated and tumor inhibition rate at day 29 was calculated. The formula of tumor inhibition rate: tumor inhibition rate(%) = [1−(baseline tumor volume of experiment group−tumor volume of experiment group at the end of experiment)/(baseline tumor volume of control group−tumor volume of control group at the end of experiment)]×100% [12]. At day 30 when the experiment ended, nude mice were executed. Tumor tissue was obtained and was fixed by 4% formaldehyde, embedded by paraffin and stained by hematoxyline and eosin (HE).

### Statistical analysis

SPSS12.0 statistical software was used. Data were shown as mean value ± standard deviation (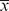
±s). Comparison between each two groups was performed by one-way ANOVA. Difference was deem statistically significant when P<0.05.

## Results

### Amplification of TNF-α gene and CD gene

**Figure 1:**
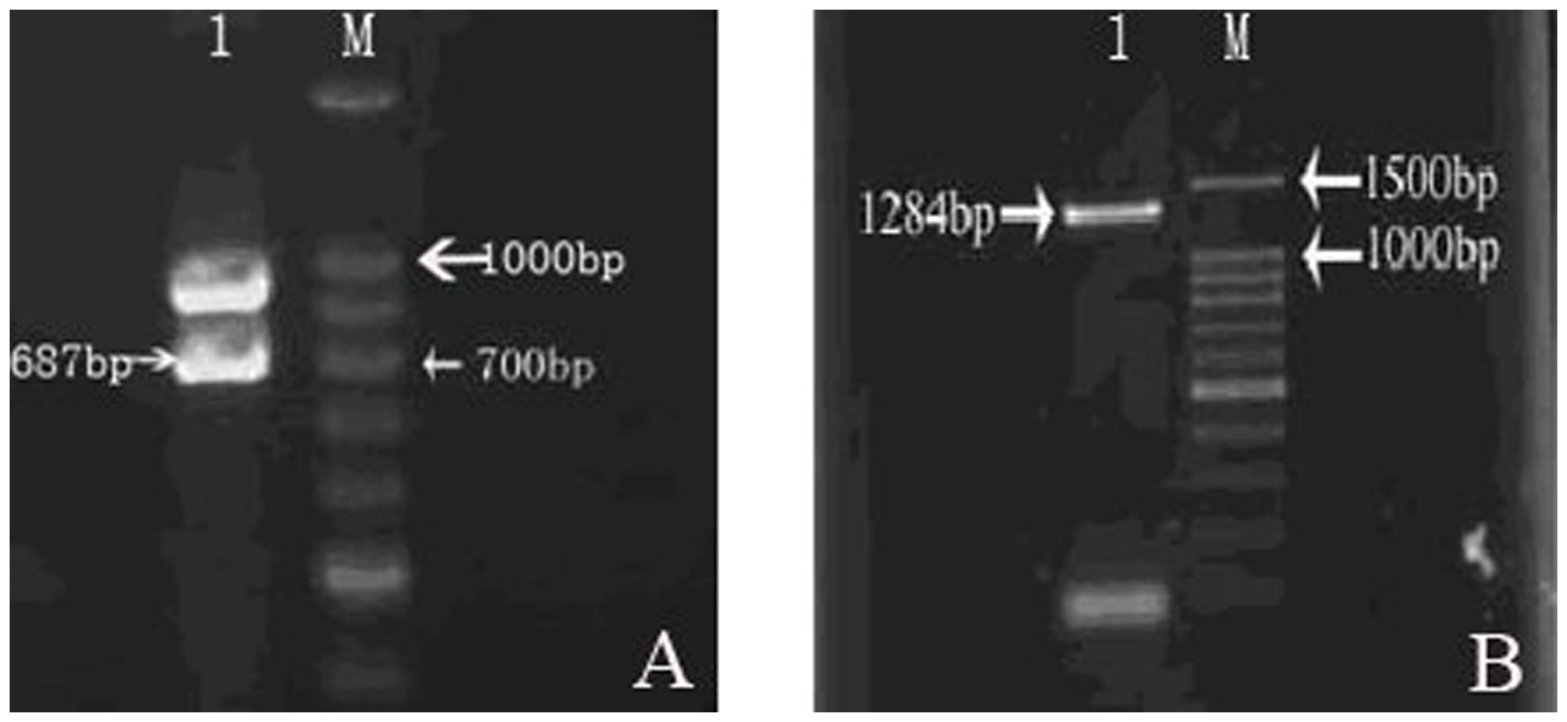
Electrophoresis analysis was performed on the RT-PCR-amplified products of TNF-α gene and revealed 700 bp amplified segments (Fig. 1 A). Whereas the amplified products of CD gene showed specific band between 1.2 kb and 1.5 kb, which was consistent with target gene in terms of gene length (Fig. 1 B) (M means DNA Marker and 1 mans PCR-amplified products of TNF-α gene in Fig. 1A; M means DNA Marker and 1 mans PCR-amplified products of CD gene in Fig. 1B).

Electrophoresis analysis was performed on the RT-PCR-amplified products of TNF-α gene and revealed 700 bp amplified segments (**Fig. 1** **A**). Whereas electrophoresis analysis on PCR-amplified products of CD gene showed specific band between 1.2 kb and 1.5 kb, which is consistent with target gene in terms of gene length (**Fig. 1** **B**).

### Verification on eukaryotic expression vectors pcDNA3.1(+)-CD, pcDNA 3.1(+)-TNF-α-IRES-CD and pcDNA 3.1(+)-TNF-α

**Figure 2:**
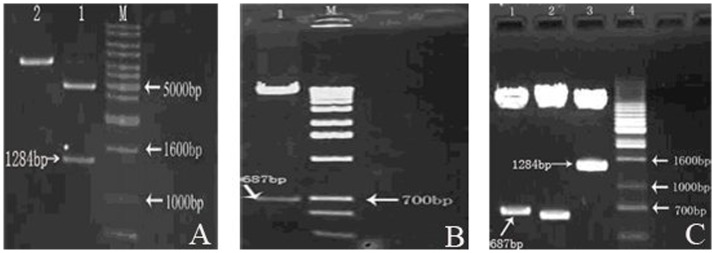
When cleavaging pcDNA3.1 (+)-CD, agarose gel electrophoresis analysis on cleavaged products revealed a band between 1.2 kb and 1.5 kb, which was consistent with target band (Fig. 2 A). When cleavaging pcDNA3.1(+)-CD -TNF-α, electrophoresis showed protein band at around 700 bp (Fig. 2 B). By cleavaging pcDNA 3.1(+)-TNF-α-IRES, respectively, TNF-α, IRES and CD were obtained (Fig. 2 C). M means DNA Marker and 1 mans pcDNA3.1(+)-CD/Xho Ι+Apa Ι in Fig. 2 A; M means DNA Marker and 1 mans pcDNA3.1(+)-CD -TNF-α/EcoR Ι+Not Ιin Fig. 2 B; 1 mans pcDNA 3.1(+)-TNF-α-IRES-CD/EcoRΙ+NotΙ, 2 means pcDNA 3.1(+)-TNF-α-IRES-CD/XhoΙ+NotΙ and 3 means pcDNA 3.1(+)-TNF-α-IRES-CD/XhoΙ+ApaΙ in Fig. 2 C.

When cleavaging pcDNA3.1 (+)-CD with double enzymes of Xho Ι and Apa Ι, agarose gel electrophoresis analysis on cleavaged products revealed a band between 1.2 kb and 1.5 kb, which was consistent with target band (**Fig. 2A**). When cleavaging pcDNA3.1(+)-CD -TNF-α with double enzymes of EcoR Ι and Not Ι, electrophoresis showed protein band at around 700 bp (**Fig. 2B**). By cleavaging pcDNA 3.1(+)-TNF-α-IRES with Xho Ι/Apa Ι, EcoR Ι/Not Ι and Xho Ι/Not Ι, respectively, TNF-α, IRES and CD were obtained (**Fig. 2** **C**).

### RT-PCR verification on the expression of TNF-α gene and CD gene

**Figure 3:**
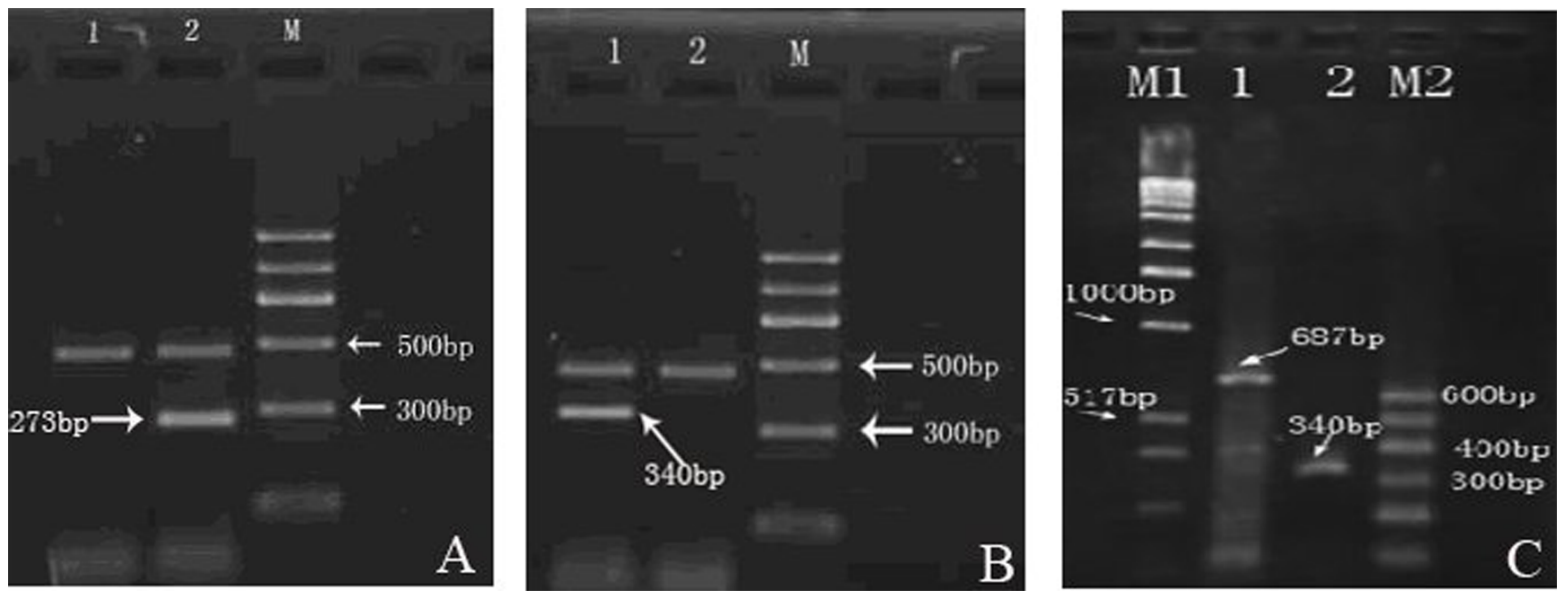
For Hep-2/0 cells which were not transfected with TNF-α gene, no noticeable TNF-α mRNA was seen (Fig. 3 A, band 1). While in transfected Hep-2/TIC and Hep-2/TNF-α cells, electrophoresis on RT-PCR products revealed specific bands of TNF-α mRNA (Fig. 3B, band 2; Fig. 3C, band 1.). In Hep-2/0 cells which were not transfected with CD gene, no mRNA of CD gene was detected (Fig. 3C, band 2). While in transfected Hep-2/TIC and Hep-2/CD cells, electrophoresis on RT-PCR products revealed specific bands of mRNA of CD gene (Fig. 3B, band 1; Fig. 3C, band 2).

For Hep-2/0 cells which were not transfected with TNF-α gene, no noticeable TNF-α mRNA can be detected (**Fig. 3** **A, band 1**). While in transfected Hep-2/TIC and Hep-2/TNF-α cells, electrophoresis on RT-PCR products revealed specific bands of TNF-α mRNA (**Fig. 3B** **, band 2;** **Fig. 3C** **, band 1**). In Hep-2/0 cells which were not transfected with CD gene, no mRNA of CD gene was detected (**Fig. 3C** **, band 2**). While in transfected Hep-2/TIC and Hep-2/CD cells, electrophoresis on RT-PCR products revealed specific bands of mRNA of CD gene (**Fig. 3B** **, band 1;** **Fig. 3C** **, band 2**).

### In vitro experiment of cytocidal effect

Hep-2/CD and Hep-2/TIC cells with positive CD gene expression were sensitive to 5-FC at the concentration above 0.01 mmol/L; the cytocidal effect increased with the concentration of 5-FC, that is, the effect was dosage-dependent. Whereas Hep-2/0 cells were hardly influenced by 5-FC below the concentration of 1 mmol/L. At the concentration level of 10 mmol/L, cytocidal effect was seen in only 20% of the cells. In Hep-2/TNF-a cells, no significant changes in cytocidal effect were found as concentration of 5-FC increased.

**Figure 4:**
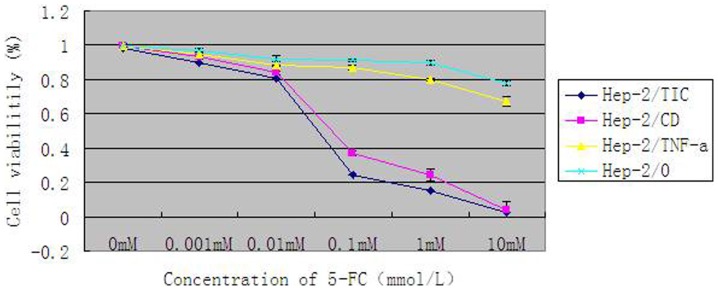
Line graph of survival rates in cells treated by 5-FC at varied concentrations for 4 days.

In Hep-2/TIC cells with concomitant expression of TNF-α and CD genes, cytocidal effect induced by 5-FC was significant higher than that in Hep-2/CD cells which merely expressed CD gene. In Hep-2/TIC and Hep-2/CD cells, half lethal dose was 0.05 mmol/L and 0.07 mmol/L, respectively. While in both Hep-2/TNF-a and Hep-2/0 cells, half lethal dose was above 10 mmol/L. 5-FC induced the strongest cytocidal effect in Hep-2/TIC cells (**Fig. 4**).

### By-stander effect in vitro

**Figure 5:**
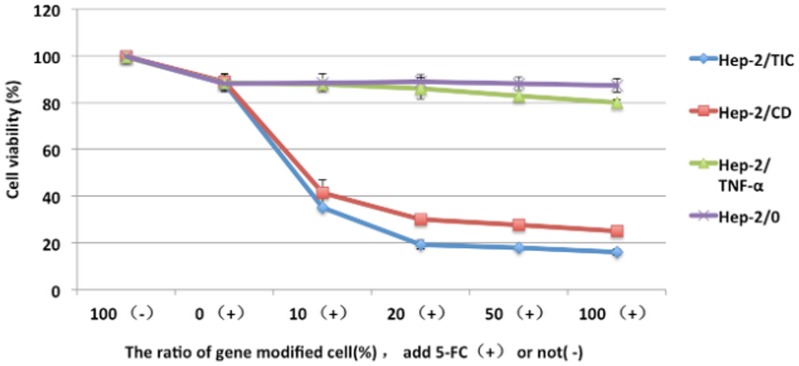
By-stander effects in varied transfected cells.

In the mixed cells in which Hep-2/TIC, Hep-2/CD, Hep-2/TNF-α and Hep-2/0 cells accounted for 20% of all cells, cell survival rates were (19.24±1.93)%, (30.07±0.95)%, (86.00±4.59)% and (88.99±2.96)%, respectively when treated by 1 mmol/L 5-FC; significant differences were seen when comparing Hep-2/TIC group to three other groups (*P*<0.05); differences between Hep-2/CD group and three other groups were also significant (*P*<0.05); however, difference was not statistically significant between Hep-2/TNF-α and Hep-2/0 groups (*P*>0.05). Cytocidal effect was seen in both Hep-2/TIC and Hep-2/CD group, more prominently in Hep-2/TIC group than in Hep-2/CD group (*P*<0.05). When Hep-2/TIC, Hep-2/CD, Hep-2/TNF-α and Hep-2/0 cells accounted for 100% of all cells and no parent cells were added, significant difference in cell survival rate was seen between each two of the four groups when treated by 1 mmol/L 5-FC (*P*<0.05). In Hep-2/TNF-α and Hep-2/0 cells, no by-stander effect was shown. When Hep-2/CD and Hep-2/TIC cells accounted for 20% of the mixed cell, cytocidal effect was seen in 70% and 80% of the mixed cells, respectively. By-stander effect was strongest in Hep-2/TIC cells when treated by 5-FC. (**Fig. 5**).

### Analysis on apoptosis

**Figure 6:**
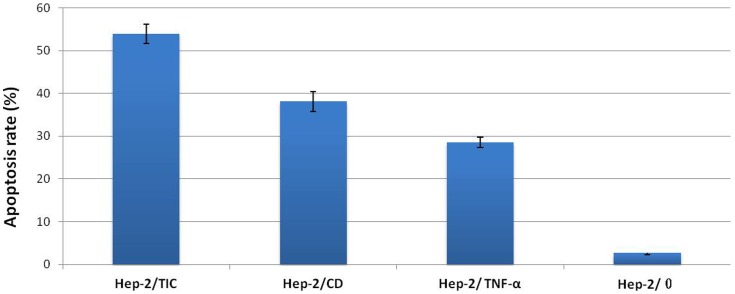
Apoptotic rates in cells treated by 5-FC for 4 days.

After being treated for 4 days, apoptotic rate was(2.73±0.42)% in Hep-2/0 group, (28.53±1.25)% in Hep-2/TNF-α group, (38.13±2.32)% in Hep-2/CD group and (53.93±2.22)% in Hep-2/TIC group; statistically significant differences were observed when comparing each two groups (*P*<0.05). Among all groups, Hep-2/TIC group showed the highest apoptotic rate. (**Fig. 6**)

### Detection of TNF-α in the supernatant of in vitro cell culture

**Figure 7:**
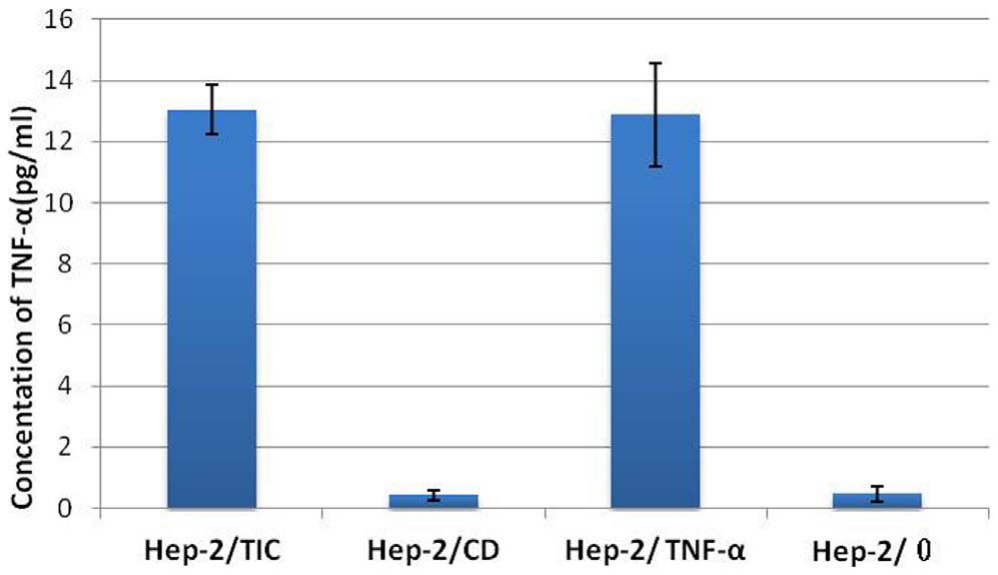
Concentrations of extracellular TNF-a protein in varied cells culture.

In Hep-2/TIC and Hep-2/TNF-α cells with positive expression of TNF-α gene, expression of TNF-α protein was detected in the supernatant of the cell culture of Hep-2/TIC and Hep-2/TNF-α. Concentrations of TNF-α protein in the supernatant of both these cell culture were significantly different as compared to that in Hep-2/CD or Hep-2/0 cells (*P*<0.05). The difference of TNF-α protein concentrations in supernatant was not significantly different between Hep-2/TIC and Hep-2/TNF-α cells (*P*>0.05); also, no significant difference was observed between Hep-2/CD and Hep-2/0 cells (*P*>0.05). (**Fig. 7** **)**.

### In vivo experiment

A classic subcutaneous inoculation method was used to establish the nude mice models of human laryngeal carcinoma. In total, tumor developed in 44 mice, with a tumor formation rate of 83%. With regard to different groups, respectively 10 mice of Hep-2/TIC group, 11 mice of Hep-2/CD group, 11 mice of Hep-2/TNF-α group and 12 mice of Hep-2/0 group developed tumor. Averagely tumor formation took 6–8 days. At day 8 after cell inoculation, diameter of the grafted tumor was around 5 cm. During the experiment, respectively 1 mouse of Hep-2/TNF-α group and Hep-2/0 group showed significant weight loss, arched back, decreased activity and listlessness at day 8 and day 10 of drug administration, respectively. They died later and were excluded from the experiment. Respectively 1 nude mouse of Hep-2/CD group and Hep-2/0 group showed tumor necrosis at day 18 and day 20 after cell inoculation and were excluded from the experiment.

### Growth curve of grafted tumor in nude mice

**Figure 8:**
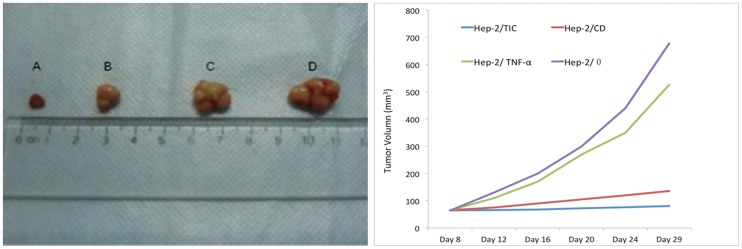
Influence of *in vivo* combined treatment of CD and TNF-a genes on the growth of grafted laryngeal carcinoma. The left picture shows the measured tumor tissues (A: Hep-2/TIC; B: Hep-2/CD; C: Hep-2/Hep-2/TNF-α; D: Hep-2/0). The right picture shows the growth curve of grafted laryngeal carcinoma.

Pictures of measured tumor tissues and growth curves of the grafted tumors in nude mice of varied groups were shown in **figure 8**. It was seen that tumor was growing fast in the nude mice of control (Hep-2/0) group; tumor growth in Hep-2/TNF-α group was hardly inhibited; tumor growth in Hep-2/CD group and Hep-2/TIC group was retarded, more prominently in Hep-2/TIC group. In the nude mice of control (Hep-2/0) group, tumor doubling time was 2.91 days; while in Hep-2/TIC group, Hep-2/CD group and Hep-2/TNF-α group, tumor doubling time was 5.34 days, 4.01 days and 2.99 days, respectively. At day 8 after cell inoculation, tumor volume was similar in Hep-2/TIC group, Hep-2/CD group, Hep-2/TNF-α group and Hep-2/0 group (64.47±4.01 mm^3^, 65.37±2.66 mm^3^, 64.84±5.41 mm^3^ and 64.02±4.78 mm^3^, respectively); no significant difference was seen between each two of them (*P*>0.05). At day 29 after cell inoculation, volume of grafted tumor in Hep-2/TIC group, Hep-2/CD group, Hep-2/TNF-α group and Hep-2/0 group was 80.47±33.74 mm^3^, 135.5±14.83 mm^3^, 525.8±40.70 mm^3^ and 678.3±52.34 mm^3^, respectively; significant difference was seen between each two of them (*P*<0.05). Tumor inhibition rates of Hep-2/TIC group, Hep-2/CD group and Hep-2/TNF-α group were 97.40%, 88.58% and 24.96%, respectively.

### Histopathology in grafted tumors

**Figure 9:**
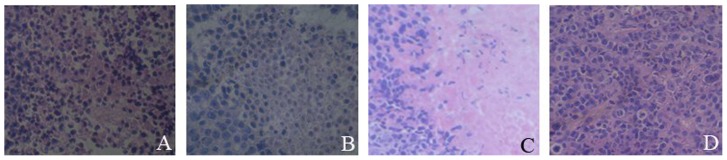
Pathological changes in the sections of varied grafted tumor tissue (Hep-2/TIC group: large amount of necrotic tumor tissue, with considerable lymphocyte infiltration and rare tumor cell division; Hep-2/CD group: considerable amount of necrotic tissue, with a few infiltrating lymphocytes; Hep-2/TNF-α group: large amount of necrotic tissue, with extremely rare infiltrating lymphocytes; Hep-2/0 group: tumor tissue of lowly differentiated squamous cell carcinoma, with frequent giant cells and dividing tumor cells, no significant tumor necrosis or lymphocyte infiltration was seen).

At day 30 post the cell inoculation, pathological changes in Hep-2/TIC group, Hep-2/CD group, Hep-2/TNF-α group and Hep-2/0 group were shown accordingly in figure 9 (A, B, C and D). Many differences can be found among the four groups. Hep-2/TIC group: large amount of necrotic tumor tissue, with considerable lymphocyte infiltration and rare tumor cell division; Hep-2/CD group: considerable amount of necrotic tissue, with a few infiltrating lymphocytes; Hep-2/TNF-α group: large amount of necrotic tissue, with extremely rare infiltrating lymphocytes; Hep-2/0 group: tumor tissue of lowly differentiated squamous cell carcinoma, with frequent giant cells and dividing tumor cells, no significant tumor necrosis or lymphocyte infiltration was seen.

## Discussion

Surgery is still the main treatment of LSCC accompanied with radiotherapy and chemotherapy in the assistant position. In addition, the improvement space of normalized surgical procedures is also limited. LSCC's recurrence and metastasis often lead to the failure of treatment and 5-year survival rate is less than 60% [5]–[6], [13]. In addition, surgery, radiotherapy and chemotherapy all have many side effects, such as part of, even complete lose of the function of larynx and serious impact on their quality of life, especially poorly target of radiotherapy and chemotherapy. Compared to previous therapy methods, gene therapy own many advantages, especially on precise target therapy and low side effects, and is becoming the most potential treatment methods of malignant tumors. Due to the occurrence, development and metastasis of the tumor are multifactorial and complex multi-stage process, so the single-gene treatment of tumor is not ideal [14]. Because tumor genesis and development are a complicated process affected by multiple factors, signal gene treatment cannot inhibit tumor survive effectively. Instead, combined gene-therapy can resolve these problems.

TNF-α is a multifunctional cytokine which has been demonstrated in numerous preclinical models with potent anticancer property, importantly, the recombinant protein is in clinical trials phase [15]–[19]. Moreover, 5-FU is one of the most popular chemotherapy drugs applying for head and neck cancer therapy and has stable and effective theraputic outcome. However, clinic application of 5-FU is limited due to the lack of target selectivity, short half-life and serious cytotoxicity. Studies have shown that the expression of local tumor suicide gene is higher 1,000 to 10,000 times higher than around organizations when treated by prodrug of CD/5-FC suicide gene, [20] CD can transform 5-FC into 5-FU which 5-FU has a better target selectivity, only inhibiting tumor cell not the normal tissue around.

In our study, both *in vivo* and *in vitro* experiments confirmed the synergic anti-tumor effect of CD gene and TNF-α gene when using in combination. Based on the therapeutic rationale of CD suicide gene/pro-drug system and TNF-α, theoretically they manage to treat laryngeal carcinoma via multiple pathways while showing additive effect: TNF-α induces high permeability of tumor vasculature and thereby helps gather and concentrate 5-FU, a chemotherapeutic agent converted from 5-FC, in the tumor tissue. In vitro experiment has demonstrated that TNF-α could accelerate the gathering of chemotherapeutic agents in certain tumor cells and help reach the half inhibitory concentration in a short period of time [21]. On the other hand, high permeability of tumor vasculature as well as high concentration of chemotherapeutic agent 5-FU render gradient concentration distribution of TNF-α in tumor tissue, rather than convective distribution, which aggregates the apoptosis of vascular endothelial cells in the tumor. The tumor cells killed by suicide gene system therapy could release antigen peptides, which could be then ingested and presented to lymphocytes by antigen present cells as activated by cytokines. Such process not only effects direct cytolysis, but also produces more cytokines and activates the production of immune cells [22]. The binding of TNF-α to the TNFR-2 on the surface of immune cells can induce a series of anti-tumor immune responses. Since nude mice have defective cellular immune, extremely few lymphocytes were seen in tumor tissue of control group of our experiment, while lymphocytes could be seen in both CD group and TNF-α group; TIC group showed most frequent lymphocytes. These findings demonstrated that CD and TNF-α induced anti-tumor immune response in synergy, while the underlying mechanisms had to be further investigated. In addition, CD gene and TNF-α gene are complementary to each other when using in combination: since transduction occurs before the therapeutic effect, the entire gene therapy has become relatively controllable, for example, when gene-transformed cells secrete excessive TNF-α, dosage of 5-FC can be regulated to allow converting from 5-FC to 5-FU (as catalyzed by CD), which then eliminates small amount of gene-transformed cells, thereby, damage to the body or adverse reactions due to overly activated immune system can be avoided [23]. The anti-tumor immune response induced by suicide gene is not strong enough, and in vivo application of suicide gene produces relatively low transduction efficiency in tumor cells, therefore it is not capable of completely eliminating the tumor; furthermore, tumors might recur when treatment stops. Whereas TNF-α can fully induce anti-tumor immune response and inhibit tumor formation and metastasis [24]. The effect of immune response and the by-stander effect of CD suicide gene are complementary and dependent to each other; the anti-tumor immune response induced by TNF-α greatly enhances the by-stander effect and extends the scope of cytocidal effect by suicide gene, and thereby overcomes the limitation of low transfection rate of suicide gene.
